# Barraquer–Simons syndrome: a rare form of acquired lipodystrophy

**DOI:** 10.1186/s13104-016-1975-9

**Published:** 2016-03-18

**Authors:** Joana Oliveira, Paula Freitas, Eva Lau, Davide Carvalho

**Affiliations:** Department of Endocrinology, Diabetes and Metabolism, Centro Hospitalar São João, Alameda Prof. Hernâni Monteiro, 4200 Porto, Portugal; Faculty of Medicine, University of Porto, Alameda Prof. Hernâni Monteiro, 4200 Porto, Portugal

**Keywords:** Acquired partial lipodystrophy, Barraquer–Simons syndrome, Cephalothoracic lipodystrophy, Adipose tissue, Hypocomplementemia

## Abstract

**Background:**

Human lipodystrophies are uncommon disorders, with important clinical consequences, which are often undiagnosed. The Barraquer–Simons syndrome is a form of partial symmetric lipodystrophy of unknown etiology, characterized by the loss of subcutaneous adipose tissue, limited to upper part of the body. Insulin resistance and metabolic complications are less common than with other lipodystrophy subtypes. Patients usually have decreased serum complement-component 3 levels, associated with complement activation by the alternative pathway, which may indicate the presence of renal involvement.

**Case presentation:**

The authors report a case of a 31-year-old woman with progressive loss of subcutaneous fat, limited to the face, neck and thorax. She presented no severe metabolic complications, neither signs of insulin resistance. Laboratory tests revealed mild dyslipidemia, and low serum levels of complement-component 3. Clinical and biochemical characteristics were consistent with the diagnosis of Barraquer–Simons syndrome.

**Conclusion:**

The present case illustrates the importance of recognizing the clinical features of this lipodystrophic syndrome, which may present potentially severe consequences and psychological distress. A brief overview is made, addressing the clinical signs of the disease, its course, and how to manage it.

## Background

Human lipodystrophies are a group of acquired or inherited disorders which are characterized by selective fat loss, ranging from partial to generalized [1, 2]. Lipodystrophies are usually tightly linked with severe metabolic complications, which highlight the significance of adipose tissue as an active endocrine organ [3]. Insulin resistance, diabetes mellitus, dyslipidemia, hypertension and hepatic steatosis are often seen in affected patients [1, 2]. The severity of metabolic disease usually correlates with the extent of fat loss, suggesting that insulin resistance could result from the absence of adipose tissue and the consequent leptin deficiency [4, 5]. More than a century ago, Mitchell [6], Barraquer [7], and Simons [8] described the first lipodystrophic disorder, known as *lipodystrophia progressiva*, or Barraquer–Simons syndrome, which is now called ‘acquired partial lipodystrophy’ (APL). Approximately 250 patients have been described in the literature [9], the majority being of European descent [10]. Females are three to four times more likely to be affected than men [11]. Fat loss usually starts during childhood or adolescence, and may follow an acute viral infection such as measles [12, 13]. Barraquer–Simons syndrome is mainly characterized by the loss of subcutaneous tissue, limited to upper part of the body, with the face, neck, arms, thorax, and upper abdomen being affected in a cephalocaudal manner (cephalothoracic lipodystrophy). In contrast, the adipose stores of the gluteal regions and lower extremities tend to be either preserved or are increased [11]. Unlike others types of lipodystrophy, insulin resistance and its related metabolic complications appear to be less frequent (diabetes 10 %, hypertriglyceridemia 30 %) and are less severe [13]. One third of patients presented membranoproliferative glomerulonephritis and associated signs of activation of alternative complement pathway—the reduction of circulating concentrations of complement-component 3 (C3), and the presence of the C3-nephritic factor [14, 15]. The authors describe the clinical case of a female patient with clinical and biochemical features consistent with Barraquer–Simons syndrome.

## Case presentation

### Case description

**Fig. 1:**
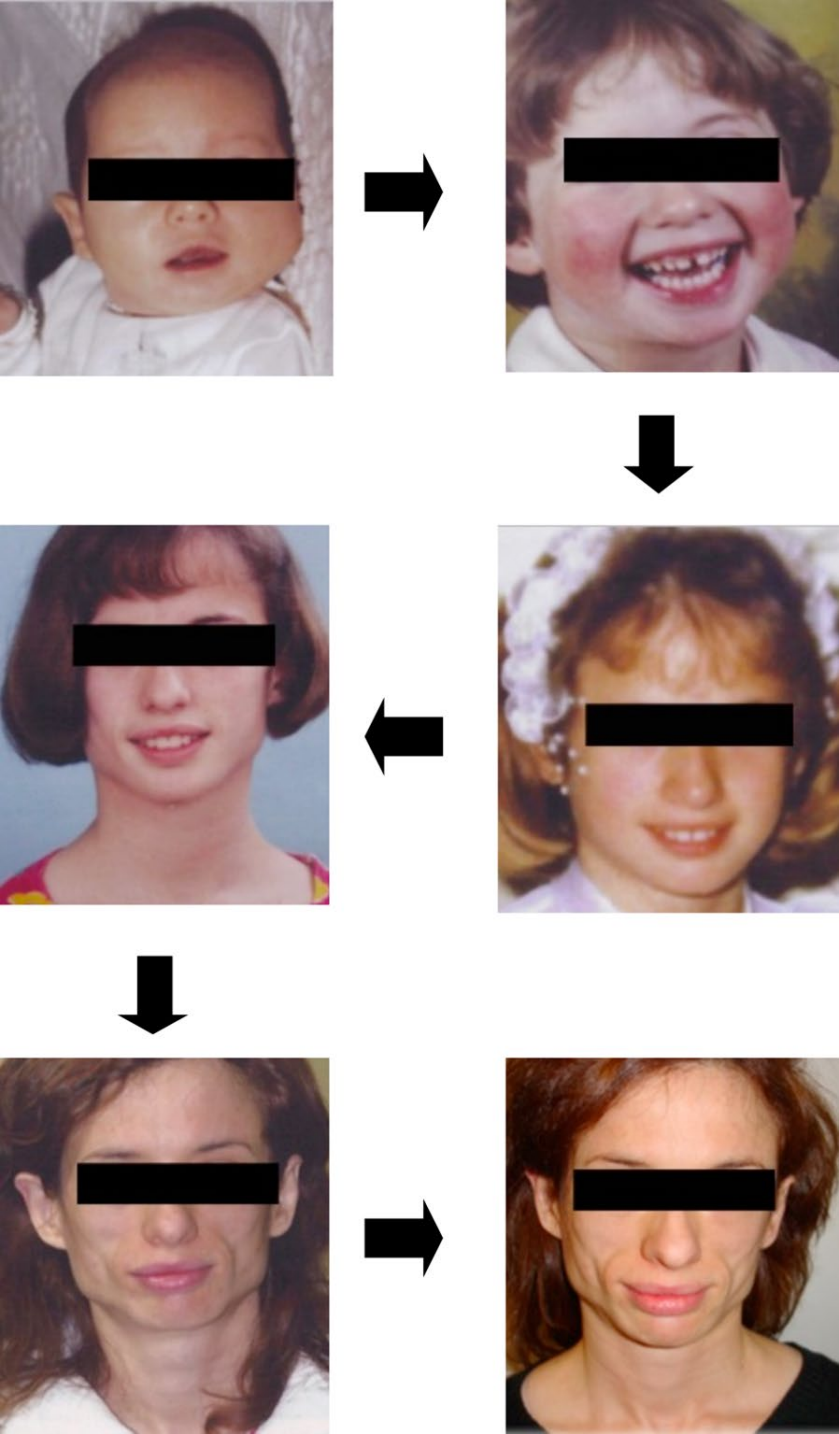
Progressive facial lipoatrophy with loss of buccal fat pads

**Fig. 2:**
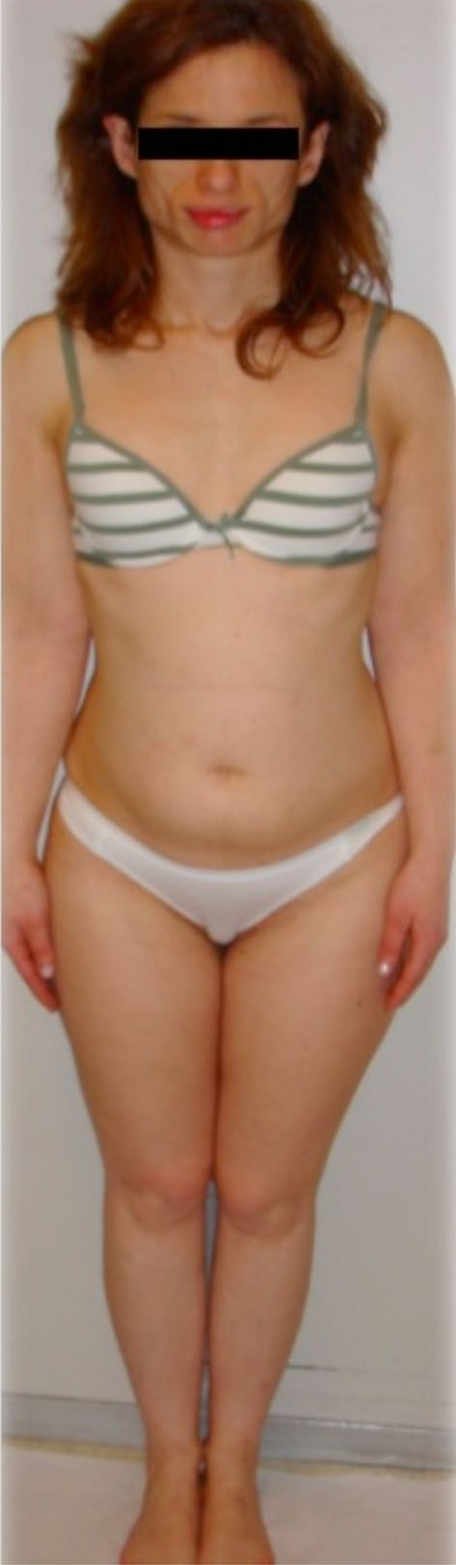
Subcutaneous adipose tissue preserved in the lower abdomen and thighs

A 31-one-year old Caucasian woman was referred to the endocrinology department for a facial lipodystrophy. She was the second child of non-consanguineous, healthy parents. The neonatal period and her psychomotor development were unremarkable. She had chickenpox during her childhood, and later mumps at the age of fifteen. Her first menstruation was when she was 12 years old, and she had had regular menstrual cycles ever since. At puberty, she noted that her subcutaneous adipose facial tissue gradually began to decrease (Fig. 1). No abnormality in her past medical history existed, and she had never used any drug that could cause lipodystrophy. There was no family history of the same condition and furthermore, she did not report any symptoms. A physical examination revealed facial lipoatrophy, with loss of buccal fat pads and prominent zygomatic arches. She presented bilateral breast hypoplasia. The subcutaneous fat was preserved in other anatomic regions, particularly in the lower abdomen and thighs (Fig. 2). She presented normal thyroid palpation. Hepatosplenomegaly, umbilical hernia, acanthosis nigricans, clitorimegaly, hirsutism or acromegalic features were all absent. Ophthalmic and other systemic examinations were unremarkable, including her neurological status and deafness was excluded. Laboratory tests, including a complete blood count, biochemical parameters (with renal and liver function tests), urine analysis with urinary albumin excretion, insulin-like growth factor-1 (IGF-1), and sexual and thyroid function tests revealed no abnormalities. Her fasting glucose (70, 82 mg/dL) and insulin (2.7 mU/mL), A1c (5.2 %) and oral glucose tolerance test (2 h plasma glucose 96 mg/dL) presented normal values. There was a discrete elevation of LDL-cholesterol (139 mg/dL), and reduced HDL-cholesterol (54 mg/dL), with normal triglycerides level. The C3 levels were low, detected as <18 mg/dL (normal: 83–177 mg/dL). The patient presented no renal disease, nor more severe metabolic disorders up until our last observation. She underwent surgical correction of facial lipoatrophy, with good aesthetic results.

## Discussion

The overall clinical and biochemical features of our patient led us to consider Barraquer–Simons syndrome as being the main diagnosis. The abnormal fat repartition was in conformity with the essential criterion proposed by Misra et al. [12]: with gradual symmetrical subcutaneous fat loss from the face, neck, upper extremities, thorax and abdomen, preserving the lower extremities. Some supportive criteria were also met, namely: onset during adolescence, the absence of a family history of lipodystrophy, and low serum levels of C3. The lack of C3 allows for a clear distinction between this syndrome and other forms of lipodystrophy. The C3-nephritic factor induces lysis of adipocytes expressing factor D (adipsin)—a serine protease enzyme—and the overt expression of factor D by numerous tissues, which produces the characteristic fat loss pattern [3, 16]. Some patients may progress to present drusen—small accumulations of hyaline bodies underneath the retina [17]. Such as in this present case, metabolic disorders can be absent in patients with late-onset partial lipodystrophy. Furthermore, acanthosis nigricans, hirsutism and signs of virilization are very unusual [13].

Mutations in several genes have been found in patients with inherited lipodystrophies, including mutations in *LMNA*, *PPARG, AKT2* and *ZMPSTE24* in partial lipodystrophy [18], and mutations in *AGPAT2, BSCL2, CAV1* and *PTRF* in congenital total lipodystrophy [19–21]. However, the molecular pathogenesis of APL has not been clearly established. In 2006, Hegele proposed that *LMNB2* could be a mutation responsible for APL. In four out of nine patients he found three new rare *LMNB2* mutations, by using candidate gene sequencing. He concluded that not all subjects with APL had *LMNB2* mutations and also found a few carrier mutations among healthy controls. Therefore, it seems that APL behaves like a complex trait, in which a susceptibility allele requires the presence of additional factors to trigger the expression of the disease [22]. A family history is usually absent, and a broad set of autoimmune diseases is often associated [17]: membranoproliferative glomerulonephritis, hypocomplementemia, systemic lupus erythematosus, dermatomyositis and localized scleroderma [23–25]. Occasional functional anomalies, such as deafness, epilepsy, and mental retardation can also be associated with the condition [26].

Therapeutic approaches for APL consist of improving esthetic appearance with plastic surgery and the management of additional systemic disorders. The main goal of cosmetic surgical procedures is to minimize the psychological discomfort that impairs the patient’s quality of life. Metabolic complications are not usually a main problem, however they still have to be screened and treated when they exist. A hypolipidic diet plan and regular exercise were advised, and medication will be offered when necessary. Thiazolidinediones stimulate growth and differentiation of adipocytes and seem to be effective in some heterogeneous forms of lipodystrophy. In 2003, Walker et al [27] reported increases of fat in buccal and subcutaneous abdominal adipose tissue in a 20-year-old woman with APL after 7 months of rosiglitazone therapy. Unfortunately, this may exacerbate the fat accumulation in non-affected regions. Recently, metreleptin, a recombinant analogue of human leptin, has been approved for the treatment of metabolic derangements of lipodystrophy. Metreleptin replaces the leptin deficiency, thus improving insulin resistance, hyperglycemia, dyslipidemia, and hepatic steatosis. Acquired partial lipodystrophy has less low leptin levels, and less metabolic derangements, and therefore metreleptin has lower efficacy [28]. The prognosis of Barraquer–Simons syndrome is mainly dependent on renal disease. A few patients have required renal transplantation for end-stage renal disease related to glomerulonephritis [29, 30].

## Conclusions

Barraquer–Simons syndrome is an extremely rare disorder with important clinical consequences and psychosocial effects. The authors underline the importance of the identification and the periodic assessment of patients with APL. Close long-term follow-up is required to identify metabolic disturbances, potentially life-threatening renal problems, and other associated diseases.

## Consent

Written informed consent was obtained from the patient for publication of this Case Report and any accompanying images.

## Abbreviations

APL: acquired partial lipodystrophy
IGF-1: insulin-like growth factor-1
C3: plasma complement-component 3
